# Large Scale Genome-Centric Metagenomic Data from the Gut Microbiome of Food-Producing Animals and Humans

**DOI:** 10.1038/s41597-022-01465-5

**Published:** 2022-06-25

**Authors:** Leandro Nascimento Lemos, Fabíola Marques de Carvalho, Fernanda Fernandes Santos, Tiago Barcelos Valiatti, Dandara Cassu Corsi, Alessandro Conrado de Oliveira Silveira, Alexandra Gerber, Ana Paula C. Guimarães, Cintya de Oliveira Souza, Danielle Murici Brasiliense, Débora de Souza Collares Maia Castelo-Branco, Eleine Kuroki Anzai, Francisco Ozório Bessa-Neto, Gláucia Morgana de Melo, Gleyce Hellen de Souza, Lúcio Fábio Caldas Ferraz, Márcia de Nazaré Miranda Bahia, Márcia Soares Mattos, Ramon Giovani Brandão da Silva, Ruanita Veiga, Simone Simionatto, Walter Aparecido Pimentel Monteiro, William Alencar de Oliveira Lima, Carlos Roberto Veiga Kiffer, Rodrigo Cayô, Ana Cristina Gales, Ana Tereza Ribeiro de Vasconcelos

**Affiliations:** 1 Bioinformatics Laboratory, National Laboratory of Scientific Computing (LNCC), Rio de Janeiro, RJ Brazil; 2 Universidade Federal de São Paulo (UNIFESP), Laboratório Alerta, Division of Infectious Diseases, Department of Internal Medicine, Escola Paulista de Medicina (EPM), São Paulo, SP Brazil; 3 Regional University of Blumenau (FURB), Blumenau, SC Brazil; 4 Seção de Bacteriologia e Micologia, Instituto Evandro Chagas (IEC), Secretaria de Vigilância em Saúde (SVS), Ministério da Saúde, Ananindeua, PA Brazil; 5 Postgraduate Program in Medical Microbiology, Group of Applied Medical Microbiology, Federal University of Ceará (UFC), Fortaleza, CE Brazil; 6 Universidade Federal de São Paulo (UNIFESP), Laboratório de Imunologia e Bacteriologia (LIB), Setor de Biologia Molecular, Microbiologia e Imunologia, Departamento de Ciências Biológicas (DCB), Instituto de Ciências Ambientais, Químicas e Farmacêuticas (ICAQF), Diadema, SP Brazil; 7 Universidade Federal da Grande Dourados (UFGD), Laboratório de Pesquisa em Ciências da Saúde, Dourados, MS Brazil; 8 Laboratory of Molecular Biology of Microorganisms, University São Francisco (USF), Bragança Paulista, SP Brazil; 9 Universidade Federal de São Paulo (UNIFESP), Laboratório Especial de Microbiologia Clínica (LEMC), Division of Infectious Diseases, Department of Internal Medicine, Escola Paulista de Medicina (EPM), São Paulo, SP Brazil

**Keywords:** Metagenomics, Food microbiology

## Abstract

The One Health concept is a global strategy to study the relationship between human and animal health and the transfer of pathogenic and non-pathogenic species between these systems. However, to the best of our knowledge, no data based on One Health genome-centric metagenomics are available in public repositories. Here, we present a dataset based on a pilot-study of 2,915 metagenome-assembled genomes (MAGs) of 107 samples from the human (N = 34), cattle (N = 28), swine (N = 15) and poultry (N = 30) gut microbiomes. Samples were collected from the five Brazilian geographical regions. Of the draft genomes, 1,273 were high-quality drafts (≥90% of completeness and ≤5% of contamination), and 1,642 were medium-quality drafts (≥50% of completeness and ≤10% of contamination). Taxonomic predictions were based on the alignment and concatenation of single-marker genes, and the most representative phyla were Bacteroidota, Firmicutes, and Proteobacteria. Many of these species represent potential pathogens that have already been described or potential new families, genera, and species with potential biotechnological applications. Analyses of this dataset will highlight discoveries about the ecology and functional role of pathogens and uncultivated Archaea and Bacteria from food-producing animals and humans. Furthermore, it also represents an opportunity to describe new species from underrepresented taxonomic groups.

## Abstract (summary)

**Table:** 

Measurement(s)	Metagenome
Technology Type(s)	Illumina Sequencing

## Background & Summary

The use of metagenomic approaches has revolutionized clinical microbiology allowing simultaneous identification of all potential pathogens without the need for culture-based methods^1,2^. For example, real-time metagenomic outbreak surveillance has also been useful in the identification and tracking of unknown infections, as such Shiga-toxigenic *Escherichia coli* (STEC) O104:H4 in Germany^3^ and SARS-CoV-2 coronavirus^4^. In the clinical context, metagenomics is also a powerful weapon in the fight against antibiotic resistance pathogens in humans and animals^5^. From the use of advanced methods based on *de novo* assembly of metagenomic sequences, several studies have reported the importance of the resistome (e.g., collection of antibiotic resistance genes)^6^. Improvement in the identification and quantification of antibiotic resistance genes from complete or near-complete genes makes the assembly approach useful for characterizing novel antibiotic resistance genes and/or comparing them with well-known genes^7^. On the other hand, it is also possible to establish the link between taxonomy and functional annotation using long-assembled sequences^8^, which can improve the characterization of antibiotic resistance genes and the identification of pathogens.

It is well known that environmental microbiomes are hotspots of antibiotic resistance genes and that these genes can be exchanged between environmental and host-associated microbiomes^6^ or between host- and host-microbiomes^9^. The One Health concept is a global strategy to study the relationship between human, animal, and environmental health. The exchange of pathogenic and non-pathogenic microorganisms among these settings, associating the interconnection between humans, animals, and the environment, has been the main focus of one health study^10^. For example, Mosites and collaborators^11^ reported that human and animal microbiomes share the same species of their gut microbiome in rural livestock-owning households in western Kenya. Another study, conducted by Sun *et al*.^11^, demonstrated that the three-month exposure of students to livestock farms resulted in high sharing of antibiotic resistance genes and the microbial community. However, to the best of our knowledge, no one health data based on large-scale sampling and high-throughput sequencing by focusing on microbial genome reconstruction from metagenome data has been available in public repositories.

Here, we present a large-scale genome-centric dataset based on a pilot-study of 2,915 metagenome-assembled genomes (MAGs) from 107 samples (Supplementary Table 4). Data can be reused to test new hypotheses about the potential exchange of microbes between food-producing animals and humans or explored in the biotechnology, evolutionary, functional, or ecological context.

## Methods

### Data generation

**Fig. 1:**
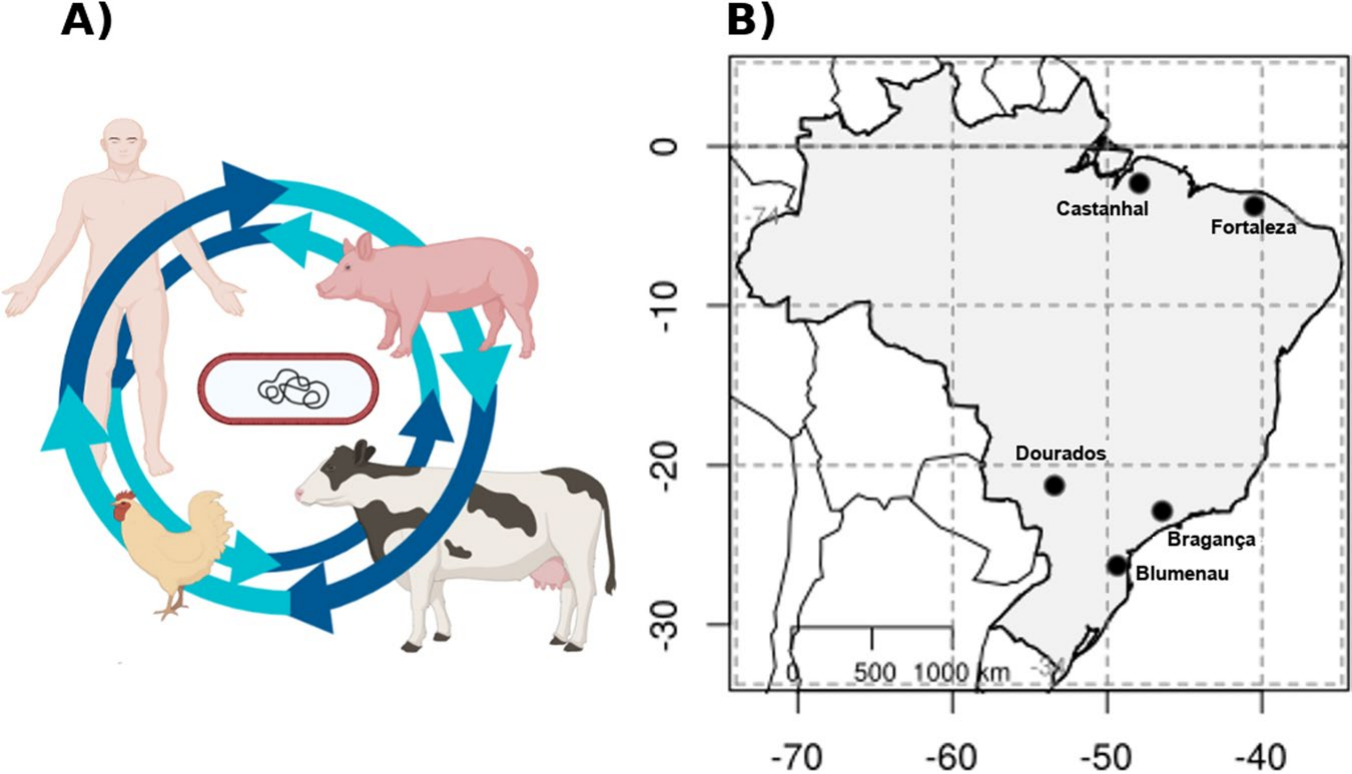
The general concept of the large-scale One Health Project and sample site locations. (**A**) Global strategy to study the relationship between human and animal health and the transfer of microbial species (pathogens and non-pathogens) between these systems. (**B**) Five geographic regions in which samples were collected in Brazil.

Data was generated from GUARANI (One Health Brazilian Group) network. Initially, the aims of the GUARANI network’s project were to quantify the abundance and diversity of antibiotic resistance genes (e.g., resistome) of a large number of samples in Brazil (South American), distributed in the major five Brazilian geographical regions (North region - Castanhal, 1°17′46.3776″ S–47°55′8.6016″ W; South Region - Blumenau, 26°55′10″ S 49°3.967′ W; Southeast Region - Bragança, 22°57′9.7″ S–46°32.651′ W; Midwest Region - Dourados, 22°13′16″–S 54°48.334′ W; Northeast Region - Fortaleza, 3°43′2″ S–38°32.584′ W), and to investigate the relationship between human and food-producing animal microbiomes, and the potential exchange of pathogenic and non-pathogenic microbes between these systems (Fig. 1A) by metagenomic approaches. Supplementary Table 1 describes information about sex, species, age of animals, and demographic localization of the farms and cities where samples were collected. In general, the experimental design was based on general and descriptive traits. To cover a high number of samples from all geographical regions of Brazil and have great potential to perform large-scale genome-centric metagenomic data, we choose to use the same samples in Illumina high-throughput sequencing. For this, 107 samples [humans (N = 34), cattle (N = 28), swine (N = 15), and poultry (N = 30)] were collected in triplicate from farms located in the five Brazilian geographical regions (Fig. 1B). For each region, properties were selected based on the criterion of simultaneous swine, poultry, and cattle rearing. Human samples were collected from healthy individuals who lived in the closest urban areas to the rural properties. The World Health Organization defines health as “complete physical, mental, and social well-being”, in this study, we followed this concept to define adults (>18y-o) without any physical disease or infirmity as healthy individuals. All human data was anonymized, and the authors affirm that human research participants provided informed consent for the publication of the microbiome data and all information was approved by the research ethics committees. Data collection was approved by the Research Ethics Committee (CEP), Committee on Ethics in the Use of Animals (CEUA) from Universidade Federal de São Paulo (UNIFESP) and National System of Genetic Resource Management and Associated Traditional Knowledge SISGEN (Process numbers: 3.116.383, 2607170119 and AA1668A, respectively). (CEP and SISGEN). All Cattle, swine, and poultry samples were collected only from adult animals. In the sample collection, a swab was introduced in the first 2 cm of the rectal region to collect faecal samples of animals. Invasive rectal swabs were used only to collect samples from animals (swine, cattle, and poultry). For humans, the subjects were instructed to collect stool samples using a sterile fecal collection container with no preservative. A sterile charcoal swab was introduced in the stool specimen, followed by the rapid removal of stool excess by pressuring the swab against the container wall. The samples were stored and shipped to a central lab for DNA extraction.

### DNA extraction and sequencing

DNA extractions were carried out under sterile conditions in a microbiological vertical laminar airflow hood. We did not use negative control samples (e.g., “blank swab”) because the reagent and laboratory contamination were most problematic in low microbial biomass microbiomes (e.g., placenta or lung human microbiome) compared that find in high microbial biomass microbiomes^12,13^, as such that found in the faecal samples used in this study. DNA was extracted directly from swabs using the ZymoBIOMICS (Zymo, USA) DNA Miniprep Kit. DNA integrity and quantification were performed using a Qubit ® 2.0 Fluorometer (Thermo Fisher Scientific, AU). All samples were quantified by Qubit and organized on the sequencing plates according to the DNA concentrations obtained (Supplementary Table 2). The samples that had the same range of amount (ng) of DNA were in the same plate, since the number of PCR cycles of amplification of the libraries depends on the amount of initial DNA, according to Illumina protocol. The samples from the different hosts were treated together with maximum attention to avoid cross contamination. In short, sequencing libraries were prepared with the Nextera DNA Flex Library Preparation Kit (Illumina, USA) according to the manufacturer’s protocol. Sequencing was carried out in the NextSeq. 500 System (Illumina, USA) using NextSeq. 500/550 High Output Kit v2.5 (300 Cycles), generating 2 × 150 bp reads.

### Pre-processing

Firstly, raw reads were removed using BBDuk software (http://jgi.doe.gov/data-and-tools/bb-tools/). Illumina adapters, PhiX and reads with Phred score below 20 were removed using the following parameters: minlength = 50, mink = 8, qout = auto, hdist = 1 k = 31, trimq = 10, qtrim = rl, ktrim = l, minavgquality = 20 and statscolumns = 5. Then, host-associated reads were also filtered using four reference genomes (*Homo sapiens* - GRCh38 v.38, *Bos taurus* - ARS-UCD 1.2, release 106_2108, *Sus scrofa* - Sscrofa 11.1, release 106_2107 and *Gallus gallus* - GRCg6a, release 104a_2108). All alignments were performed in Bowtie 2.4.1 using the very-sensitive options^14^.

### Metagenome assembly, binning, and genome quality control

**Fig. 2:**
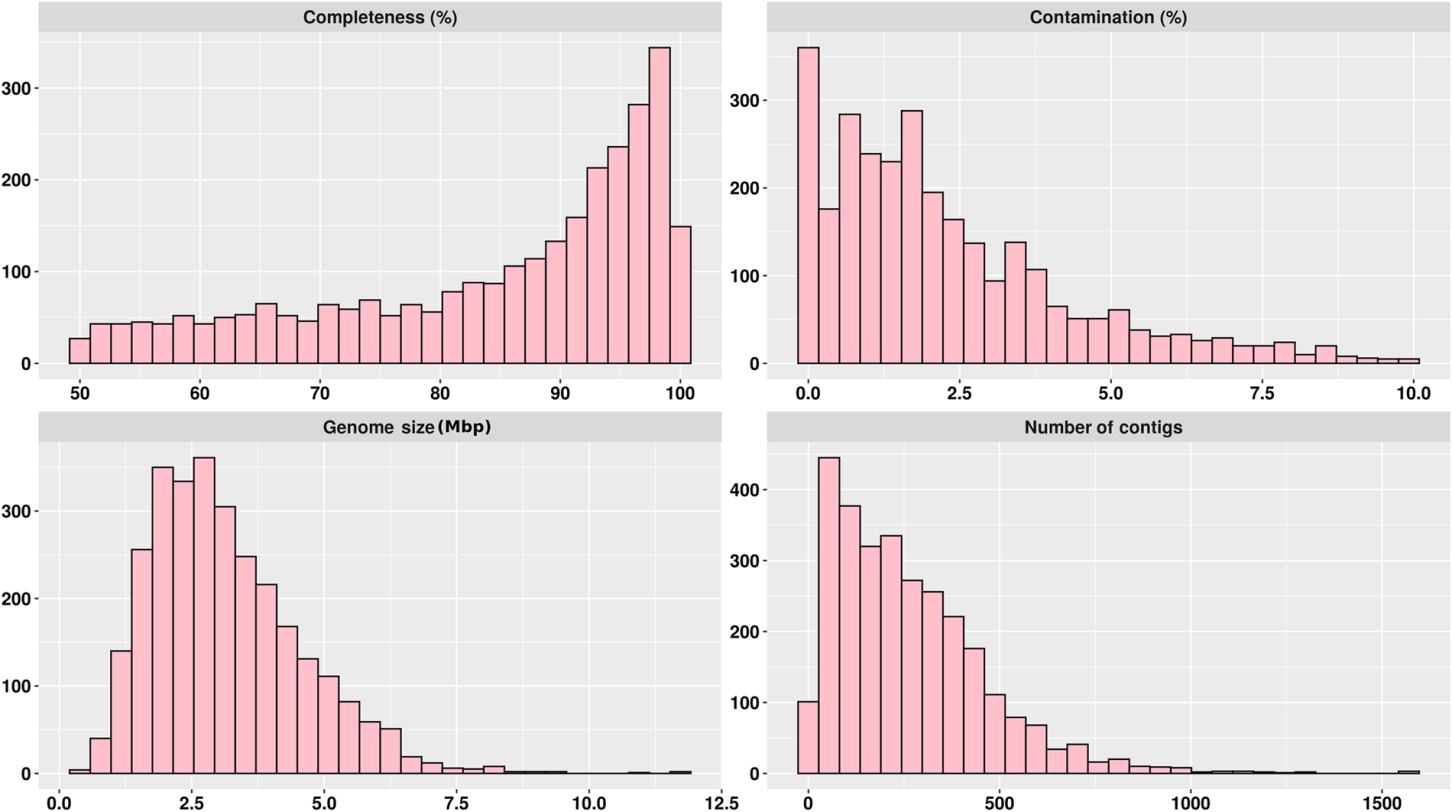
Quality determination of metagenome-assembled genomes (MAGs). (**A**) Completeness and (**B**) contamination were estimated by the identification of individual marker genes. (**C**) Genome size was calculated by the sum of bases present in all contigs of each MAG. (**D**) Number of contigs.

To increase the throughput and maximize the number of MAGs in this dataset, we choose a strategy based on co-assembly. This strategy has been used in several studies, including in the reconstruction of genomes from poultry^15^, cattle^16^, and human^17^ metagenomes. In this case, samples were merged using the combination of host and region samples (See Supplementary Table 3 to check each Co-assembly dataset). Metagenomes were assembled using Megahit software^18^ with the meta-large option (–min-count 2–k-list 27,37,47,57,67,77,87). A total of 4,861,910,960 high-quality reads were used to assemble 1,676,286 contigs greater than 2,500 bp (Table 1). The binning approach was used to reconstruct genomes from metagenomes based on the compositional traits of individual contigs (e.g., tetra-nucleotide frequency and coverage) using Metabat2 with default parameters^19^. We considered only the genomes that passed rigorous quality control to remove spurious and contaminated genomes in the downstream analyses. Genomes with completeness ≥50.0 and contamination ≤10.0 were used in the downstream analyses, following the Minimum Information about a Metagenome-Assembled Genome (MIMAG) of bacteria and archaea standards^20^ in CheckM software^21^ with CheckM (lineage workflow). A total of 2,915 MAGs were reconstructed (Table 2 and Supplementary Table 3). Of these MAGs, 1,273 are high-quality drafts (≥90% of completeness and ≤5% of contamination), and 1,642 are medium-quality drafts (≥50% of completeness and ≤10% of contamination) (Fig. 2). The mean and standard deviation of genome size were 3.1 ± 1.4 Mbp, while the number of contigs had a mean of 263 ± 263. In addition, the mean genome size is compatible with those described in human stool communities^22^. On the other hand, we assembled contigs greater than 2.02 Mbp in MAGs from poultry metagenomes, indicating the accuracy of the metagenome assembly. All MAGs were submitted under the NCBI database and post-processing through NCBI’s Contamination Screen to remove adaptador and cross-species contamination.

**Table 1:** Number of reads and metagenome assembly metrics of each individual data set.

Host	Region	Number of samples	Number of high-quality reads	Number of assembled contigs	Number of Contigs (≥2,500 bp)	Total length of sequence ≥2,500 bp	Longest contig (bp)
**Human (N** = **34)**	Castanhal	6	259,016,108	1,634,409	81,182	670,841,654	1,057,294
	Bragança	7	310,616,922	2,255,248	119,571	970,361,522	1,034,121
	Blumenau	7	296,871,652	1,703,918	93,700	756,020,767	838,472
	Dourados	7	284,108,138	1,491,995	85,735	719,165,690	1,187,438
	Fortaleza	7	446,738,660	2,216,926	133,034	1,171,492,042	1,168,256
	**Total**	34	—	—			—
**Cattle (N** = **28)**	Castanhal	6	254,494,314	2,220,977	75,955	678,070,566	1,234,574
	Bragança	6	360,597,786	4,093,158	160,163	1,127,539,900	1,105,705
	Blumenau	6	300,801,100	3,666,305	137,492	1,017,974,954	1,436,631
	Dourados	6	237,490,610	2,509,355	93,416	792,840,322	891,031
	Fortaleza	4	150,991,730	1,892,645	69,144	535,215,175	981,474
	**Total**	**28**	—	—			—
**Swine (N** = **15)**	Castanhal	3	117,694,018	1,158,246	48,717	437,660,170	788,418
	Bragança	3	117,387,524	1,077,507	38,045	385,280,349	980,111
	Blumenau	3	151,335,286	1,643,937	74,497	613,584,395	1,004,084
	Dourados	3	123,568,764	1,331,505	53,338	420,121,709	1,376,043
	Fortaleza	3	169,768,812	1,391,433	60,545	587,776,398	826,244
	**Total**	**15**	—	—			—
**Poultry (N** = **30)**	Castanhal	6	311,668,528	1,974,410	98,496	848,169,168	978,697
	Bragança	6	141,138,814	1,140,082	47,610	459,976,910	690,014
	Blumenau	6	223,399,652	1,296,760	62,518	640,029,927	2,020,273
	Dourados	6	226,320,474	1,598,993	70,086	668,128,667	1,234,552
	Fortaleza	6	377,902,068	1,457,250	73,042	730,871,569	831,481
	**Total**	**30**	—	—			—

**Table 2:** Number and quality of metagenome-assembled genomes (MAGs) of each individual dataset.

Host	Region	Number of samples	Number of Genomes (MAGs)^1^	Medium-quality (MAGs)^2^	High-quality (MAGs)^3^
**Human (N** = **34)**	Castanhal	6	131	68	63
	Bragança	7	219	128	91
	Blumenau	7	167	91	76
	Dourados	7	153	87	66
	Fortaleza	7	294	164	130
	**Total**	**34**	**964**	**538**	**426**
**Cattle (N** = **28)**	Castanhal	6	138	75	63
	Bragança	6	183	134	49
	Blumenau	6	183	111	72
	Dourados	6	146	86	60
	Fortaleza	4	117	73	44
	**Total**	**28**	**767**	**479**	**288**
**Swine (N** = **15)**	Castanhal	3	80	36	44
	Bragança	3	75	32	43
	Blumenau	3	112	62	50
	Dourados	3	109	60	49
	Fortaleza	3	147	77	70
	**Total**	**15**	**523**	**267**	**256**
**Poultry (N** = **30)**	Castanhal	6	148	96	52
	Bragança	6	90	42	48
	Blumenau	6	124	62	62
	Dourados	6	141	73	68
	Fortaleza	6	158	85	73
	**Total**	**30**	**661**	**358**	**303**

^1^Genomes with completeness => 50.00 and contamination = <10.00 ^2^Genomes with completeness = >50.00 and = <90.00 and contamination = <10.00; ^3^Genomes with completeness = >90.00 and contamination = <5.00.

### Taxonomy prediction

**Fig. 3:**
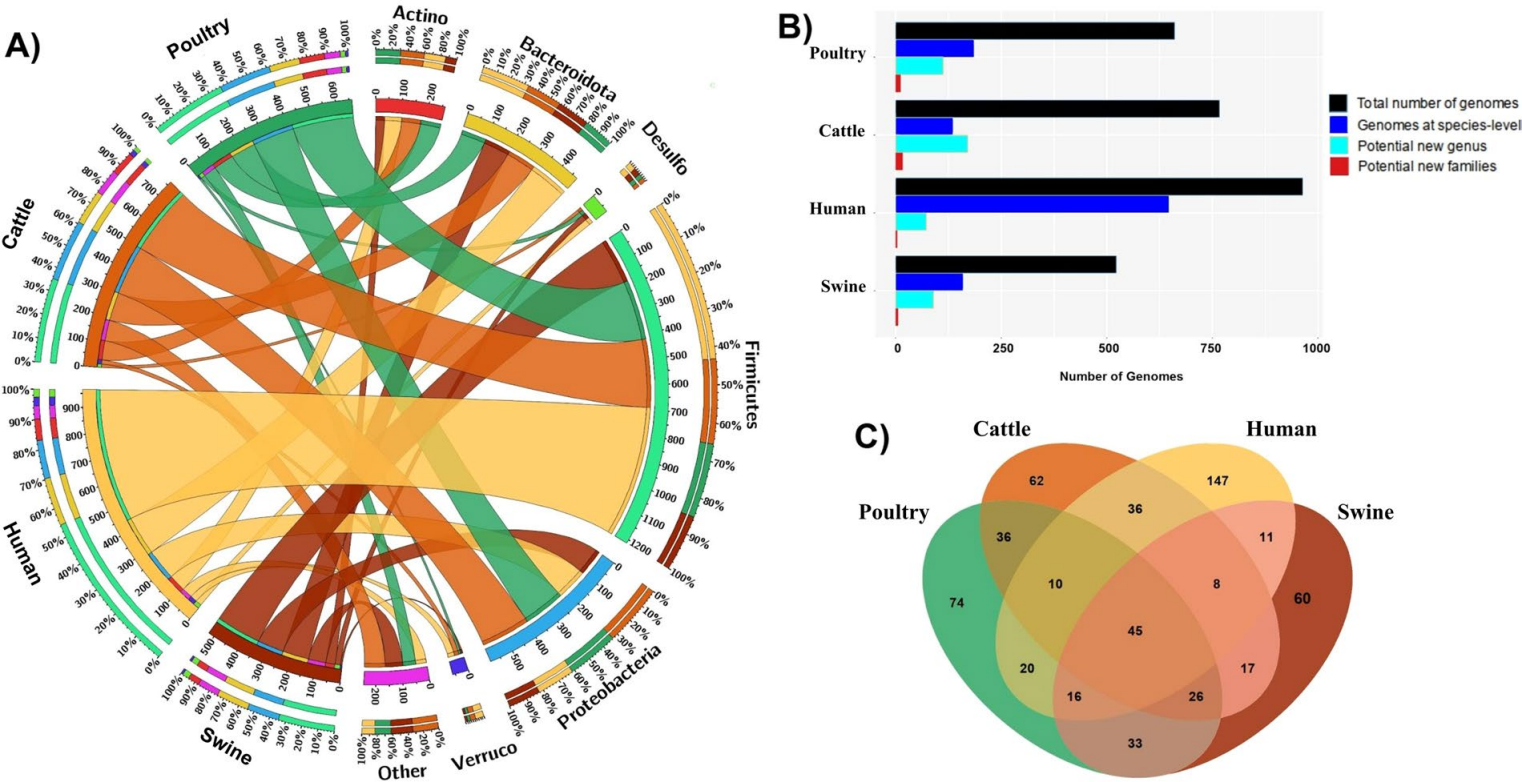
Taxonomy and host-distribution of MAGs. (**A**) Circus plot demonstrating the abundance of phyla in each host microbiome. The external track indicates the relative number (%) of phyla or host. The internal track shows the absolute number of MAGs generalized by phyla or host, (**B**) Number of potential novel lineages for each host microbiome, and (**C**) Absolute number of shared MAGs assigned to the genus level between host-associated microbiomes (human, swine, cattle, and poultry).

We used standardized bacterial taxonomy based on genome phylogenomics proposed by Parks and collaborators^23^, using the GTDB-Tk v1.3.0 software^24^ (classify_wf workflow) and the most recent version of the Genome Taxonomy Database (GTDB) Release 05-RS95^23^. This workflow has been used to infer the taxonomy of MAGs, once improved classification of new uncultivated lineages and standardized taxonomy ranks based on the phylogenetic information. The most representative phyla were Firmicutes, Bacteroidota, and Proteobacteria (Fig. 3A), which are extensively studied in host-associated microbiomes^25^. However, many of the MAGs described here are potential new genera or new families (Fig. 3B), highlighting new insights about the ecophysiology of these new taxonomic groups. Regarding shared species between the four microbial community hosts, 45 genera were shared among distinct hosts (Fig. 3C – Supplementary Table 5). This includes environmental species with ecological importance in the digestive microbiomes (e.g., *Cellulomonas* and *Azospirillum*). Furthermore, four shared genera were generically assigned as SZUA-444, SZUA-584, UBA1305, and UBA8346, demonstrating the importance of this dataset to explore new taxonomic groups.

## Data Records

The Whole Genome Shotgun project (PRJNA682348)^26^ has been deposited at DDBJ/ENA/GenBank under the accessions JAEVYR000000000-JAEWNV000000000, JAEWNW000000000-JAEXCD000000000, JAEXCE000000000-JAEXRH000000000, JAEXRI000000000-JAEYGM000000000, JAEYGN000000000-JAEYNF000000000. JAEYNG000000000-JAEZCI000000000, JAEZCJ000000000-JAEZRM000000000 and JAEZRN000000000-JAFAGR000000000 (Supplementary Table 3 - NCBI Genome Accession column). The raw data of Illumina metagenomic sequencing reads was deposited in SRA-NCBI (www.ncbi.nlm.nih.gov/sra) under Bioproject accession PRJNA684454^27^.

## Technical Validation

Here, we reported 2,915 draft genomes assembled from host-associated metagenomes. Illumina metagenomic reads used to assemble MAGs went through multiple steps of rigorous quality control, which included removing low-quality reads and host-associated sequences. Only a small proportion of the reads (14.64 ± 11.19%) were removed during the quality control, which had 0.22 ± 2.12% of host-associated reads (Supplementary Table 2). In a total, 4,861,910,960 high-quality reads were used in the downstream analyses.

A total of 37,755,059 contigs were generated during the metagenome assembly steps, being 1,676,286 contigs greater than 2,500 bp were assembled (Table 1). Small contigs (≲2,500 bp) were discarded because they carried less compositional signatures (as such used in the binning step: tetranucleotide frequencies and coverage) and can bias the construction of clusters during the metagenome-assembled genomes reconstruction step^28^. The longest contigs showed a mean of 1,083,245 ± 295,772 bp (max: 2,020,273; min: 690,014), demonstrating the effectiveness of the high sequencing depths used here. These results are similar to those already described in other studies reconstructed contigs greater than 900,000 bp using host-associated microbiomes like rumen metagenomes^29^ or caecum chicken microbiome^15^.

Each metagenome-assembled genome (MAG) was validated using the rigorous standards defined by the Minimum Information about a Metagenome-Assembled Genome (MIMAG) of bacteria and archaea consortium^20^, considering only medium and good quality genomes assigned by the number of single-copy genes within a phylogenetic lineage^21^. Furthermore, only 33 (1.13% of the total dataset) MAGs showed adaptor or cross-species contaminations during the NCBI’s Contamination Screen, demonstrating the high quality of this dataset. As shown in the previous section, the biological traits (e.g., genome size and the number of contigs of each mags) were similar to those recently reported in human, poultry, swine, and cattle stool communities, demonstrating that the genomes showed good quality and can be used by the scientific community to generate new studies.

## Supplementary information

Supplementary Table 1. Information about the sex, species, strain and ages of animals used to generated data showed here.

Supplementary Table 2. DNA Quantification of individual samples used in this study

Supplementary Table 3. Sequencing quality of control of all samples and co-assembly datasets used in this study.

Supplementary Table 4. Genome features of the metagenome-assembled genomes (MAGs) described in this study.

Supplementary Table 5. Shared microbial genomes in genus level among Human, Poultry, Swine, and Cattle microbiomes

## Data Availability

All software used in this study was published in peer-reviewed journals. Additional information was described in detail in the Material and Methods section
